# P-1144. Low prevalence of neonatal sepsis due to Streptococcus agalactiae in two large Mexican NICUs: multicenter retrospective study from 2021-2023

**DOI:** 10.1093/ofid/ofae631.1331

**Published:** 2025-01-29

**Authors:** Ana Ballesteros-Suarez, Abraham P Garza-Castro, Lindsay A Concha-Mora, Ericka Paulina Prieto-Baca, Oscar Tamez-Rivera, Misael Salazar-Alejo

**Affiliations:** Departamento de Medicina, Tecnológico de Monterrey, Escuela de Medicina y Ciencias de la Salud, Monterrey, México, Monterrey, Nuevo Leon, Mexico; Residencia de Pediatría, Programa Multicéntrico de Especialidades Médicas. ITESM-SSNL. Tecnológico de Monterrey, Escuela de Medicina y Ciencias de Salud., Santiago, Nuevo Leon, Mexico; Residencia de Pediatría, Programa Multicéntrico de Especialidades Médicas. ITESM-SSNL. Tecnológico de Monterrey, Escuela de Medicina y Ciencias de Salud., Santiago, Nuevo Leon, Mexico; Departamento de Medicina, Tecnológico de Monterrey, Escuela de Medicina y Ciencias de la Salud, Monterrey, México, Monterrey, Nuevo Leon, Mexico; Tecnologico de Monterrey, Escuela de Medicina y Ciencias de la Salud, Monterrey, Nuevo Leon, Mexico; Departamento de Medicina, Tecnológico de Monterrey, Escuela de Medicina y Ciencias de la Salud, Monterrey, México, Monterrey, Nuevo Leon, Mexico

## Abstract

**Background:**

Group B *Streptococcus* (GBS), or *S. agalactiae,* is reported as a leading cause of neonatal sepsis in several regions including the United States. Although in recent years its incidence has shown a steady decline, current treatment guidelines still consider it a predominant cause of neonatal sepsis. Its prevalence varies according to geographical and racial factors; however, information regarding the epidemiology and clinical presentation of neonatal GBS infection in Mexico is scarce.

**Figure:**
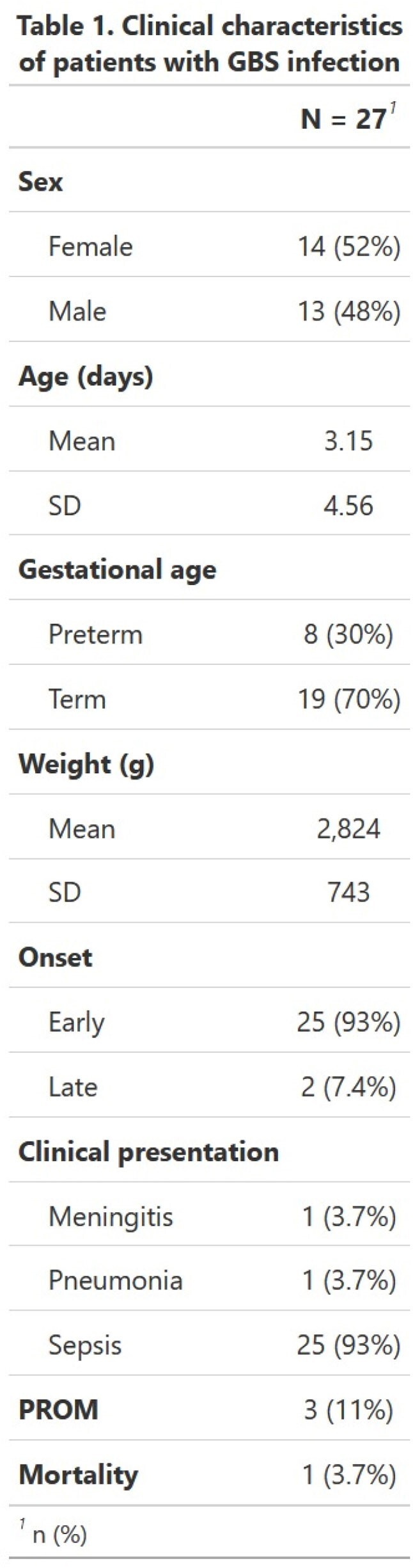

**Methods:**

Retrospective multicenter study conducted in the NICUs of the two pediatric reference hospitals in NL, Mexico. Data of all newborns ≤28 days (d) diagnosed with blood culture-proven GBS infection from 2021-2023 were collected. Maternal premature rupture of membranes (PROM) was defined as >12h. GBS infection was classified as early (< 7 d) and late-onset (≥7 d). LBWIs were defined as newborns < 2500 g and very LBWI (VLBWI) as < 1500 g at birth, respectively. Clinical scenarios were classified as sepsis, pneumonia and meningitis. Sepsis was classified as early (≤3 d) and late-onset ( >3 d). Descriptive statistical analyses were performed to determine frequencies.

**Results:**

A total of 928 positive blood cultures were included. 28 blood cultures (3%) from 27 patients were positive for GBS. Maternal PROM was documented in 11.1%. Most patients were term infants (77%) and 92.6% of the cases were early-onset. The most common presentation was sepsis (92.6%). One patient (3.7%) had pneumonia and one (3.7%) had meningitis due to GBS. Low mortality was documented (3.7%). Table 1 shows clinical characteristics of patients with GBS infection. As expected, all isolates were susceptible to Ampicillin.

**Conclusion:**

We report a low prevalence of culture-proven GBS infection (3%) in two large Mexican NICUs during the studied period. This is consistent with previous single-center reports from Mexico, which date back to more than two decades. Although the international epidemiology and treatment guidelines consider GBS a predominant cause of neonatal sepsis, its frequency in Mexico remains low. The vast majority of cases were early-onset infections, and the most common clinical presentation was sepsis, which is consistent with global epidemiology.

**Disclosures:**

**All Authors**: No reported disclosures

